# Expert consensus recommendations for the prevention and management of immunoglobulin therapy-related adverse reactions in primary and secondary immunodeficiencies: a national Delphi study from the Immunodeficiency Group of the Spanish Society of Immunology

**DOI:** 10.3389/fimmu.2026.1901301

**Published:** 2026-08-06

**Authors:** Andrea Ferranti-Ramos, Marta Aguilar-Criado, Carmen L. Avendaño-Monje, María de las Mercedes Díaz Luna, Cinthya M. Fusi-Velarde, Jaime Pons

**Affiliations:** 1 Immunology Genetics Unit, Complejo Hospitalario Universitario de Cáceres, Cáceres, Spain; 2 Department of Immunology and Genetics, Complejo Hospitalario Universitario de Badajoz, Badajoz, Spain; 3 Department of Immunology, Complejo Asistencial Universitario de León, León, Spain; 4 Primary Immunodeficiency Unit, Internal Medicine Department, Hospital Universitario y Politécnico La Fe, Valencia, Spain; 5 Research Group of Chronic Diseases and HIV Infection, Health Research Institute La Fe, Valencia, Spain; 6 Immunology Department, Hospital General Universitario Dr. Balmis, Alicante, Spain; 7 Immunology Department, Hospital Universitario Son Espases, Palma, Spain; 8 Health Research Institute of the Balearic Islands (IdISBa), Palma, Spain; 9 University of the Balearic Islands (UIB), Palma, Spain

**Keywords:** adverse drug reactions, anaphylaxis, Delphi consensus, immunoglobulin therapy, infusion reactions, intravenous immunoglobulin, premedication, subcutaneous immunoglobulin

## Abstract

**Introduction:**

Human immunoglobulin (IG) therapy, administered intravenously (IVIG) or subcutaneously (SCIG), is a cornerstone treatment for primary and secondary immunodeficiencies (PID and SID) and is increasingly used in autoimmune and inflammatory diseases. Although generally safe, adverse drug reactions (ADR) may affect treatment adherence, patient safety, and quality of care. Practical recommendations for the prevention and management of IG-associated ADR remain limited.

**Objective:**

To develop expert-based recommendations for the prevention, monitoring, classification, and management of ADR associated with IVIG and SCIG therapy in patients with PID and SID.

**Methods:**

A modified Delphi consensus was conducted by the Immunodeficiency Group of the Spanish Society of Immunology (GISEI). An expert coordinating committee developed 44 preliminary statements based on scientific evidence and clinical experience. Statements covered eight domains, including administration practices, ADR classification, premedication, acute management, complementary studies, anti-IgA antibodies, and SCIG-related issues. A national panel of specialists evaluated each statement using a 6-point Likert scale. Consensus was defined as ≥75% agreement.

**Results:**

Forty-two recommendations achieved consensus and were included in the final document. High agreement was reached regarding individualized risk assessment, infusion rate adjustment, patient monitoring, and early ADR management. Most IVIG-related ADR were considered mild, occurring during or shortly after infusion, and generally not requiring treatment discontinuation. Premedication was recommended only for selected high-risk patients. SCIG was recognized as a safe and effective alternative associated with fewer systemic reactions and predominantly mild local ADR. The panel also highlighted the limited utility of serial anti-IgA antibody testing and emphasized patient education and periodic retraining for home-based SCIG administration.

**Discussion:**

This Delphi-based consensus provides practical and clinically applicable recommendations for the prevention and management of ADR associated with IVIG and SCIG therapy. These recommendations may contribute to standardizing clinical practice, improving patient safety, optimizing treatment tolerability, and reducing variability in IG administration across healthcare settings. Further prospective studies are needed to better define risk factors and preventive strategies for severe ADR associated with IG therapy.

## 1 Introduction

Human immunoglobulin (IG) therapy, administered intravenously (IVIG) or subcutaneously (SCIG), represents a cornerstone treatment for inborn errors of immunity, also referred to as primary immunodeficiencies (PID) (1), secondary immunodeficiencies (SID), and a wide range of autoimmune, neurological, and inflammatory diseases, either as replacement therapy or for immunomodulation.

Over recent decades, IVIG and SCIG have demonstrated comparable efficacy (2, 3), differing primarily in their pharmacokinetic and safety profiles, thereby allowing for individualized treatment (4). In particular, SCIG has become established as an effective and safe alternative by providing more stable IgG levels, fewer systemic adverse drug reactions (ADR), and greater patient autonomy (5).

The favorable safety profile of current IG products is the result of decades of pharmaceutical refinement. Since the first therapeutic use of IG in 1952, manufacturing processes have undergone continuous optimization to enhance product safety, stability, and clinical tolerability. Initial preparations were limited by the presence of IgG aggregates capable of activating the complement system, resulting in frequent infusion-related ADR.

During the early 1960s, manufacturing strategies incorporating enzymatic digestion and chemical modification were implemented to reduce anticomplementary activity. The subsequent development of second-generation IVIG products, based on diethylaminoethyl (DEAE) anion-exchange chromatography, represented a major advance by enabling efficient purification while preserving the structural and functional integrity of IgG molecules. Early IVIG formulations were predominantly lyophilized to provide prolonged storage stability, typically for 2–3 years. In the mid-1980s, the introduction of low-pH formulations (approximately pH 4.25) further improved product stability and contributed to enhanced viral safety. Modern IVIG products are manufactured using sophisticated fractionation, purification, and pathogen-reduction technologies, yielding highly purified, iso-osmolar, low-sodium preparations formulated with safer stabilizing excipients. Collectively, these technological advances have markedly improved the safety profile of IVIG while preserving its efficacy for both IG replacement and immunomodulatory indications (6).

Despite these advances, ADR may still occur with variable incidence depending on the product, patient population, and route of administration (2). ADR associated with IG therapy have a multifactorial origin and may be related to product characteristics, administration-related factors, and patient-specific conditions (3). In this context, individualized risk assessment, adequate hydration, and close monitoring during infusion are essential to reduce ADR (2–4).

ADR may be classified according to their timing and severity, facilitating their recognition and clinical management. Most are mild or moderate, transient, and reversible, commonly presenting as headache, fever, chills, myalgia, or cutaneous reactions during or shortly after infusion (2). In contrast, severe ADR are uncommon but clinically significant and include anaphylaxis, thromboembolic events, acute kidney injury, aseptic meningitis, hemolysis, and transfusion-related acute lung injury (TRALI), potentially requiring hospital management (5).

Although international recommendations regarding IG therapy are available, these guidelines mainly focus on diagnostic and therapeutic aspects and do not specifically address the prevention and practical management of infusion-related ADR (7, 8).

In Spain, the absence of a unified national protocol contributes to heterogeneous clinical practices among centers. To address this need, the Immunodeficiency Group of the Spanish Society of Immunology (GISEI) promoted a nationwide Delphi consensus involving experts in PID and SID management from across the country. The aim of this document is to establish practical recommendations to improve patient safety and quality of care in the management of ADR associated with IG therapy.

This manuscript constitutes a recommendation guideline based on expert consensus from GISEI and the available evidence. Its objective is to standardize clinical practice, optimize the prevention and early recognition of ADR, guide their therapeutic management, and promote patient safety and quality of care during IVIG and SCIG treatment. These recommendations should be applied individually according to patient characteristics and clinical context.

## 2 Materials and methods

### 2.1 Study design and expert panel

A set of recommendations (R) was developed using a modified Delphi methodology. A GISEI coordinating committee defined the scope of the document and drafted the initial statements based on the available evidence and clinical experience in the management of ADRs associated with IVIG and SCIG. The consensus process was conducted at a national level, with the participation of specialists experienced in the use of IG therapy from different fields and regions across Spain, representing the main specialties involved in the management of primary and secondary immunodeficiencies and immunoglobulin therapy. All panelists were selected based on their recognized expertise in the field and had more than 10 years of clinical experience in the use of immunoglobulin therapy (**Table 1**).

**Table 1:** Clinical specialty and geographic distribution of the experts participating in the Delphi panel.

Spanish autonomous community	Immunologists	Pediatricians	Internists	Allergists
Madrid	4	2		
Catalonia	2	3		
Valencian Community	2		1	
Extremadura	3			
Balearic Islands	1	1		
Castile and León	1			
Cantabria	2			
Canary Islands				1
Andalusia			1	
Region of Murcia	1			
Total	16	6	2	1

### 2.2 Development of statements and consensus process

The 44 preliminary statements were organized into thematic blocks covering IG administration, classification and management of ADR, premedication, complementary tests, anti-IgA antibodies, and subcutaneous (SC) administration. Each statement was rated using a 6-point Likert scale. Consensus was defined as at least 75% of responses being “mostly agree” or “strongly agree”. Statements reaching this threshold were included in the document as formal recommendations. This cut-off was selected in accordance with commonly accepted thresholds used in Delphi studies in healthcare research, where agreement levels ranging from 70% to 80% are generally considered indicative of consensus (9).

Statements that did not reach consensus in the first round were reviewed by the coordinating committee, refined based on panelists’ comments, and submitted for a second voting round. Of the statements re-evaluated in the second round, all but two achieved the predefined consensus threshold and were incorporated into the final set of recommendations. The two statements that did not reach consensus in either round are included in this document for transparency, explicitly noted as having failed to achieve consensus and therefore not endorsed as formal recommendations by the panel. A third Delphi round was considered unnecessary because most statements had already achieved consensus after the first round, whereas the remaining statements showed only minor changes in the level of agreement after the second round. Therefore, additional rounds were considered unlikely to substantially modify the results and might have increased participant fatigue and attrition. This approach is consistent with modified Delphi methodologies, in which two rounds are frequently considered sufficient when stability of responses has been achieved.

### 2.3 Statistical analysis and final document

The analysis of responses was descriptive, with calculation of response frequencies and percentages, as well as the overall percentage of agreement for each item. The final version included 42 recommendations, accompanied where necessary by comments based on the available evidence and the clinical consensus of the group.

### 2.4 Ethics statement

This study was conducted as an expert consensus exercise using a modified Delphi methodology. No individual patient data were collected, no clinical interventions were performed on human subjects, and no biological samples were obtained. In accordance with applicable national and institutional regulations, this type of consensus study does not require formal approval by an ethics committee or institutional review board. All participants were informed of the purpose and scope of the study and provided their implicit consent by participating in the consensus process.

## 3 Results

A total of 42 recommendations achieved consensus and are presented in **Supplementary Table 1**, along with their corresponding levels of agreement. The following sections provide a detailed description of each recommendation and its supporting rationale, organized by thematic domain.

### 3.1 General considerations regarding the administration of IG

R1. Before the administration of IVIG or SCIG, all patients must be informed about the characteristics of the treatment and possible ADR. They must receive clear instructions on when to contact the medical center if they experience post-infusion symptoms (96% consensus).

The administration of IVIG and SCIG must be preceded by a structured process of informing the patient about the indication, benefits, possible ADR, and recommended actions in the event of symptoms during or after the infusion. This process is considered essential for treatment safety, patient understanding, and early detection of ADR, especially in higher-risk patients (10–13).

While the panel evaluated the process of informing the patient prior to treatment rather than the use of a written consent form, obtaining and documenting informed consent is considered good clinical practice; its specific format and documentation should follow each institution’s policies and applicable national regulations.

R2. The use of IVIG and SCIG should be assessed on an individualized basis for each patient, considering different factors, including comorbidities (renal failure, heart failure, hypertension, diabetes mellitus, etc.) (96% consensus).

Guidelines recommend individualizing the indication, regimen, and administration of IG, as some ADR are more frequent during first administrations or after prolonged intervals between doses. In IVIG, the infusion rate should be adjusted according to risk factors, comorbidities, and previous tolerance. In addition, for both IVIG and SCIG, renal function assessment and adequate hydration are recommended to reduce renal and thrombotic complications (10, 11, 13).

Hemolysis is another recognized, although uncommon, complication of IVIG, caused by the passive transfer of anti-A and anti-B isohemagglutinins contained in the product; the risk is greatest in recipients of non-O blood group receiving high-dose IVIG, particularly in the context of an underlying inflammatory state (14).

R3. In patients receiving IVIG for the first time, the infusion should always be started at the lowest possible rate, even when premedication has been administered. Subsequently, the rate may be increased gradually and in a controlled manner, according to the patient’s clinical tolerance and without ever exceeding the maximum recommended rate for the product (92% consensus).

Guidelines recommend initiating the first IVIG infusion at the minimum rate, especially in treatment-naïve patients or after switching products. Many acute ADR are associated with rapid administration and can be prevented by a slow initiation. In addition, each commercial preparation has specific infusion rate recommendations (15).

Although premedication may reduce some ADR, it does not replace cautious administration. Guidelines consider it a complementary measure while maintaining slow initiation of the first infusion as a basic safety recommendation. Once good tolerance has been confirmed, the administration rate may be progressively increased in subsequent cycles (10, 11, 13).

Active or intercurrent infection at the time of infusion has been identified as a predisposing factor for a higher frequency of ADR (16); in this setting, a slower infusion rate and closer clinical monitoring are advisable. Some experts have additionally proposed administering a reduced dose during the first infusion to improve tolerability, although this practice is based on clinical experience rather than on controlled evidence.

### 3.2 General considerations and classification of ADR

R4. ADR such as facial erythema, fever, chills, asthenia, general malaise, mild headache, and pruritus are considered mild ADR (88% consensus).

ADR associated with IG are classified according to severity as mild, moderate, or severe. Mild immediate reactions are the most frequent, occurring in approximately 1–15% of infusions (17), and include headache, fever, chills, asthenia, and general malaise (2, 4). They are usually transient, low in intensity, and appear during or within the first hours after infusion, generally related to the rate of administration and improving when the infusion rate is reduced (2, 3).

This classification facilitates monitoring in outpatient settings and, according to expert panels, supports that these reactions do not contraindicate IVIG administration (18), reinforcing its overall safety profile.

R5. ADR such as urticaria, wheezing, myalgias, vomiting, chest pain, lower back pain, dizziness, arthralgias, nausea, hypertension, or severe headache are considered moderate ADR (84% consensus).

Available data show that a relevant proportion of ADR associated with IG therapy correspond to moderate forms. Among the most frequent manifestations are headache, fever, and nausea, accounting for approximately one quarter or more of the ADR described in some studies (19, 20).

R6. ADR such as hypotension, bronchospasm, anaphylaxis, aseptic meningitis, thromboembolic events, acute renal failure, TRALI, or alterations in mental status are considered severe ADR (92% consensus).

Severe ADR associated with IVIG are uncommon (less than 5%) (18), although they may be potentially life-threatening, and include anaphylaxis, bronchospasm, hypotension, thromboembolic events, acute renal failure, and TRALI (3, 8). Manifestations such as aseptic meningitis and neurological alterations have also been described, although they are infrequent (21). Aseptic meningitis shows variability in its classification and may be considered moderate or severe depending on its clinical presentation and course (3, 22).

R7. Most ADR to IVIG are mild, occur during the infusion or within the first 24 hours, and do not constitute a contraindication to continuing treatment (92% consensus).

ADR associated with IG usually occur during the infusion or within the first hours afterward, especially during the first 30 minutes and, in most cases, within the first 72 hours (18, 19). They generally do not contraindicate continuation of treatment, as they can usually be resolved with simple measures such as reducing the infusion rate or temporarily interrupting the infusion, with overall good tolerability also being observed (4, 18, 21, 23).

R8. Moderate and severe ADR are uncommon or exceptional (96% consensus).

Moderate and severe ADR associated with IVIG, such as anaphylaxis, thromboembolic events, or renal failure are rare or exceptional (17, 23). Pharmacovigilance data confirm this low incidence and show that mild ADR clearly predominates (2, 22).

R9. Infusion rate, high dosage, higher concentration of the gammaglobulin preparation, and the absence of premedication are factors that may increase the risk of ADR (88% consensus).

Various factors related to IVIG administration are associated with an increased risk of ADR, with infusion rate being one of the most relevant. An increase in infusion rate is associated with greater frequency and severity of reactions, whereas reducing the rate facilitates their control (18, 23, 24). Administration of high doses, particularly immunomodulatory regimens (≥1 g/kg, typically ranging from 1 to 3 g/kg per cycle), has also been associated with an increased risk of ADR, including complications such as aseptic meningitis (24).

Regarding concentration, commercially available IVIG preparations are formulated at 5% or 10%, the 10% being the most widely used; preparations of higher concentration (16.5–20%) correspond to SC, not IV formulations. Current evidence does not consistently demonstrate a higher ADR risk for 10% compared with 5% IVIG; rather, other characteristics of the preparation, such as osmolality or excipients, may influence tolerability, especially in patients with comorbidities (23, 25). The absence of preventive measures, such as premedication or optimization of the infusion process, may also increase the risk of these events (2).

### 3.3 Premedication

R10.- In patients initiating treatment with IVIG or SCIG, the indication for premedication should be individualized and based on clinical risk factors, medical history, and the care setting (94% consensus).

The systematic administration of premedication, defined as the routine use of antihistamines, antipyretics, or corticosteroids before each IVIG/SCIG cycle, has been a widespread practice in some clinical settings, despite the limited evidence supporting its efficacy (4).

A retrospective study in patients with Kawasaki disease found that, although 97% of patients received premedication prior to IVIG infusion, the ADR rate remained at 26%, a figure comparable to that reported without systematic premedication (20%) (26). This finding suggests that premedication did not produce a significant reduction in ADR compared to the expected baseline risk.

Current clinical practice promotes an individualized approach tailored to the patient’s prior experience, avoiding unnecessary generalizations.

R11.- In patients with a history of ADR, risk factors, or comorbidities that increase the likelihood of an ADR, premedication may be prescribed from the beginning (94% consensus).

Several studies and systematic reviews have identified certain subgroups of patients who may benefit more clearly from preventive pharmacological measures before infusion.

Among the most important risk factors are a history of moderate or severe ADR to previous IVIG/SCIG infusions, particularly anaphylaxis (1). Other studies agree that the highest risk occurs during the first IVIG administration, which is why some authors recommend close monitoring at this stage (15, 16). Patients with relevant comorbidities should also be considered as potential candidates for premedication, including those with renal insufficiency, cardiovascular disease, thrombotic history, or those receiving high doses of IVIG, in whom ADR may have a greater clinical impact (3, 27). In these cases, premedication could reduce the likelihood of clinical exacerbation secondary to ADR or, at least, improve overall treatment tolerability. This individualized approach, supported by the literature, seeks to maximize treatment safety without incurring unnecessary exposure to additional medications or increasing the risk of associated complications (28).

Furthermore, as discussed in R24, in patients with PID or SID receiving IG as replacement therapy who present recurrent or clinically significant systemic ADR to IVIG, transition to SCIG should be considered as a preventive alternative when the underlying indication permits subcutaneous replacement.

R12. The use of premedication and monitoring of possible ADR during IVIG administration should be protocolized in hospital centers where it is administered regularly (88% consensus).

IVIG administration requires a structured clinical setting that ensures active monitoring, especially during the first infusions or in patients with risk factors (18). It is recommended to systematically record data such as batch number, manufacturer, dose, and infusion rate, as well as to have written institutional protocols on premedication, follow-up, and management of ADR (29, 30).

R13. Premedication should be administered at least 30 minutes before starting the IVIG infusion (88% consensus).

Premedication prior to IVIG infusion should be administered sufficiently in advance to ensure effective plasma concentrations at the time of exposure. Several studies recommend a minimum interval of 30 minutes to optimize the prophylactic effect, especially considering that many ADR occur during the first minutes of infusion (4, 18, 31).

R14. Paracetamol (acetaminophen) is, in most cases, the first therapeutic step in patients requiring premedication before IVIG administration (79% consensus).

Paracetamol is considered the first step in the premedication of patients receiving IVIG within a symptomatic and stepwise approach to ADR management, reserving antihistamines or corticosteroids for more severe cases (32). Its favorable safety profile, good tolerability, and low risk of ADR or drug interactions make it a preferred option compared with other medications (33, 34).

R15. The recommended dose of paracetamol as premedication is 1 gram, administered orally or intravenously (88% consensus).

Several studies indicate that a single 1-gram dose of paracetamol, administered orally or intravenously (IV), is effective for the symptomatic management of mild ADR associated with IVIG, such as fever, headache, or myalgias (35, 36). Although there is no universally established standard dose, 1 g is the most used dose in Spanish hospitals protocols because of its balance between efficacy and safety.

R16. Premedication with H1 antihistamines (such as dexchlorpheniramine or diphenhydramine) is recommended in patients with a history of rash, pruritus, or urticaria associated with previous IVIG administration (84% consensus).

Premedication with H1 antihistamines is indicated in patients with a history of mild cutaneous reactions associated with IVIG, such as pruritus, urticaria, or skin rash (30, 35). Available evidence supports their preventive use in patients with a history of infusion reactions, especially those with prior cutaneous involvement, and this is also a common practice in clinical settings (4, 25, 32, 37).

R17. The recommended premedication dose of dexchlorpheniramine is 5 mg IV (or the equivalent according to the specific drug or the corresponding weight-based dose in children) (88% consensus).

Dexchlorpheniramine is one of the H1 antihistamines mostly used as premedication in patients with a history of cutaneous reactions associated with IVIG. In adults, the usual dose is 5 mg IV, based on clinical experience and hospital protocols, whereas in the pediatric population, the dose should be adjusted according to body weight (32, 35, 37, 38).

R18. Premedication with corticosteroids should be reserved for cases with previous moderate or severe ADR (92% consensus).

Routine use of corticosteroids as premedication is not recommended in all patients. Available evidence supports their usefulness mainly in patients with a history of moderate or severe ADR or with a higher risk of complications, whereas their systematic use is not justified. In addition, their indication should be individualized because of the potential risk of ADR, especially in immunocompromised patients (3, 31, 39, 40).

R19. For corticosteroid premedication, hydrocortisone 75 mg, dexamethasone 4 mg IV, or methylprednisolone 40 mg IV are preferred, or the corresponding weight-based doses in children (88% consensus).

When corticosteroid premedication is indicated, the most frequently used agents are hydrocortisone, methylprednisolone, and dexamethasone (2, 28). The doses typically used are consistent with the pharmacological equivalences among systemic corticosteroids, although there is no evidence demonstrating the superiority of one over another. Therefore, the choice should be individualized according to patient characteristics and clinical experience (41).

R20. In patients with risk factors or a history of moderate or severe ADR, premedication with combinations of paracetamol, H1 antihistamines, and corticosteroids is preferable to monotherapy (84% consensus).

Combined premedication targets multiple pathophysiological pathways simultaneously, which theoretically enhances its preventive effect compared to monotherapy. While this approach is widely adopted in clinical practice and some observational studies suggest a protective effect on treatment tolerability, the evidence base remains limited and largely derived from non-controlled studies. Until stronger evidence is available, the combination regimen is therefore preferred in patients at higher risk or with a prior history of moderate to severe ADR (2, 25, 32, 33).

R21.- The need for premedication and the drugs used should be adjusted according to the patient’s tolerance and may be progressively reduced until completely withdrawn if no associated risk factors are present (88% consensus).

When a patient has adequately tolerated previous IVIG cycles without ADR, the risk of developing new reactions decreases significantly, which justifies reconsidering the need to maintain premedication on a continuous basis. Guo et al. (2) described that ADR are more common during the first administrations and tend to diminish with successive infusions, particularly in the absence of changes in dose or product formulation. This finding is further supported by the results of Kato et al. (42), who observed that patients who did not experience ADR in previous cycles maintained good tolerance in subsequent infusions, suggesting that the need for premedication can be reassessed on a case-by-case basis.

**Figure 1:**
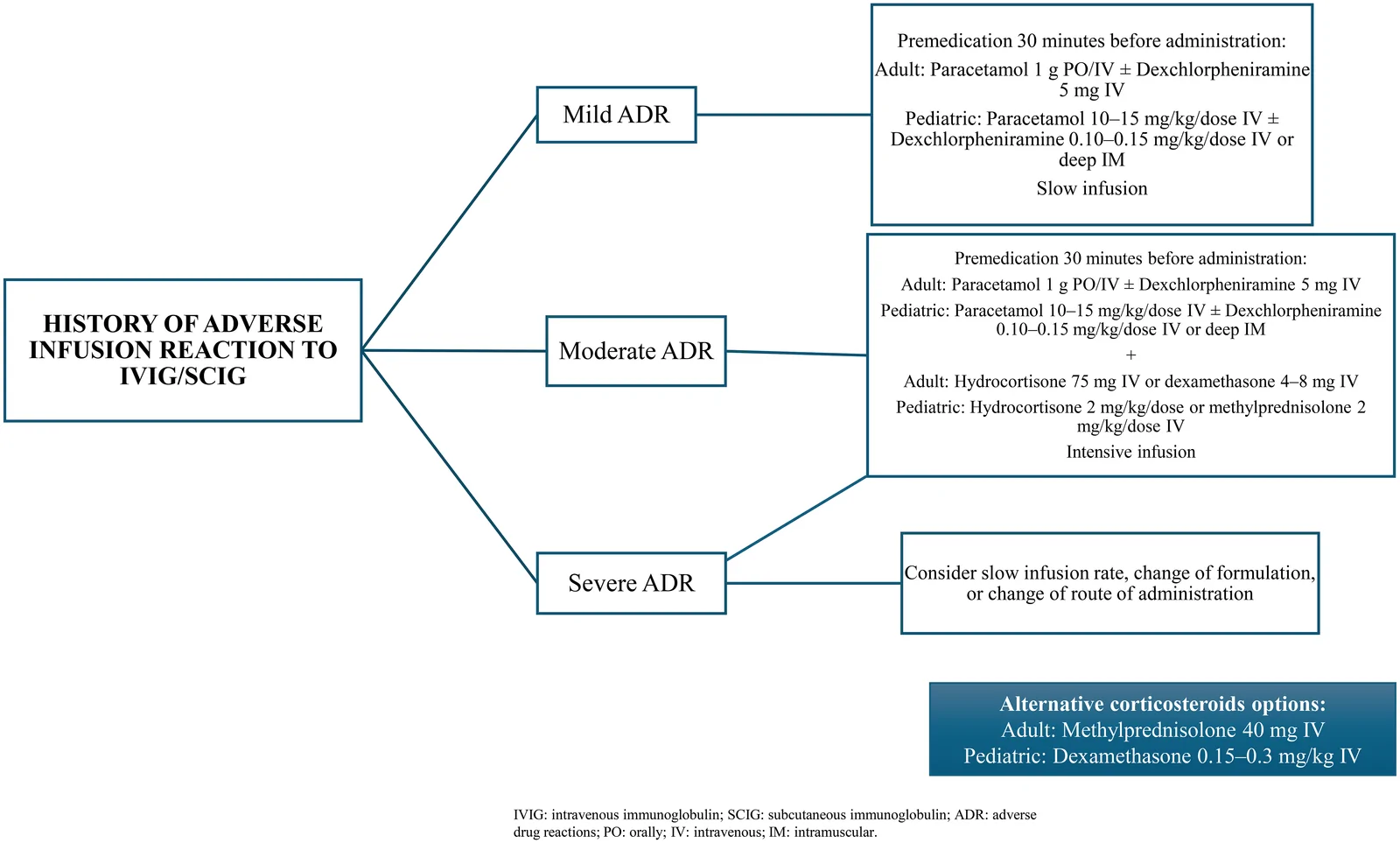
Practical algorithm for premedication decision-making in patients with a history of ADR to IVIG/SCIG, stratified by severity of previous reactions. Adult and pediatric doses are specified for each recommended agent.

The premedication recommendations described in this section (R13–R20) are summarized in **Figure 1**, which presents a practical algorithm for premedication decision-making based on the severity of previous ADR. The algorithm integrates the individualized approach endorsed by the panel, distinguishing between mild, moderate, and severe reactions and specifying the recommended agents, doses, and routes of administration for both adult and pediatric patients.

### 3.4 General management of ADR

R22. Although there are no major differences in IVIG-associated ADR among different brands or formulations, switching products without a justified reason is not recommended to preserve treatment tolerability and avoid an unnecessary increase in the risk of ADR (80% consensus).

IVIG preparations differ in aspects such as stabilizers, osmolality, or residual IgA content, factors that may influence individual tolerability and the risk of ADR, especially in patients with comorbidities (39). Formulations containing sugars usually have higher osmolality and a greater risk of nephrotoxicity, particularly in patients with renal risk factors. Therefore, in frail patients or those at risk of nephrological complications, preparations stabilized with amino acids, such as glycine or proline, are generally preferred because of their better renal tolerability (2, 3). In addition, maintaining the same preparation in stable patients with good tolerability may reduce the risk of ADR; therefore, several consensus documents and the European Medicines Agency discourage unnecessary product switching (4, 43, 44).

R23. Switching IVIG preparations should be reserved for patients with previous ADR (84% consensus).

Switching preparations should be reserved for patients with previous ADR or justified situations, such as supply shortages or specific clinical indications. In these cases, closer monitoring during the first infusions after the switch is recommended (2).

R24. In patients who do not tolerate the administration of any IVIG preparation, the use of SCIG is a good therapeutic option to maintain treatment (96% consensus).

In patients with poor tolerance to the IV route, SCIG constitutes an effective and safe alternative, associated with a lower risk of systemic ADR and better long-term tolerability. In addition, it improves quality of life by allowing home self-administration.

Beyond patients who do not tolerate any IVIG preparation, transition to SCIG should also be considered as a preventive strategy in patients with PID or SID receiving replacement therapy who present recurrent or clinically significant systemic ADR to IVIG or who have risk factors or comorbidities increasing the likelihood of ADR, provided the underlying indication permits SC replacement (e.g., PID). In indications requiring high-dose immunomodulation, the feasibility of switching may be more limited (5, 45).

R25. Monitoring during the first two hours of infusion should be maintained even if the patient has received premedication (96% consensus).

Most immediate ADR occur during the first two hours of infusion; therefore, monitoring during this period is essential (3, 4). In a prospective study involving 1, 765 infusions, the incidence of immediate ADR was 2.15%, with a mean onset time of 85.7 minutes (median 60) from the start of the infusion (15, 16). Monitoring should include periodic assessment of vital signs and surveillance for symptoms such as headache, chills, chest pain, or general malaise, even in patients who have received premedication. In addition, progressive adjustment of the infusion rate and adequate hydration are recommended (42, 46).

### 3.5 Immediate treatment of ADR

R26. Reducing the infusion rate constitutes the first measure in the management of mild ADR (92% consensus).

Symptoms such as chills, fever, back pain, or headache have been associated with the infusion rate of IVIG. Therefore, when mild ADR occurs, reducing the infusion rate constitutes an effective measure in most cases (2, 15, 27).

R27. Treatment with paracetamol and/or antihistamines constitutes the first therapeutic step in mild ADR and, in most cases, is sufficient (100% consensus).

If reducing the infusion rate does not control mild ADR, temporary interruption of the infusion and reassessment of the patient after 15–30 minutes are recommended, considering the administration of symptomatic treatment such as paracetamol, analgesics, or antihistamines (47). If symptoms resolve and vital signs are normal, the infusion may be restarted at a lower rate with close monitoring (44).

Headache associated with IG infusion is usually controlled with paracetamol or non-steroidal anti-inflammatory drugs such as ibuprofen. In adults, the usual recommendation is paracetamol 1 g IV or ibuprofen 400 mg, whereas in the pediatric population, doses should be adjusted according to body weight (3, 4, 21).

In the event of a cutaneous hypersensitivity reaction associated with IG, administration of IV antihistamines is recommended (3, 4). Dexchlorpheniramine, 5 mg IV or intramuscular (IM) in adults, with weight-adjusted dosing in children, is one of the most commonly used options.

In persistent local reactions, after mechanical measures such as gentle massage or cold or warm compresses, analgesics such as paracetamol or ibuprofen or topical treatments such as diphenhydramine or corticosteroids may be used (4).

R28. In the event of a severe ADR to IVIG infusion, administration should be stopped immediately, adequate IV hydration should be ensured, and symptomatic treatment should be initiated, which may include corticosteroids, antihistamines, analgesics, or epinephrine, as indicated. In addition, transfer or admission to a hospital should be considered depending on the patient’s clinical course (100% consensus).

Management of severe ADR should begin with immediate discontinuation of the infusion and monitoring of vital signs (4). Pharmacological treatment includes IV corticosteroids and antihistamines, with doses adjusted according to the patient’s age and body weight. Many severe ADR resolve with these measures, although some cases may require additional treatments such as epinephrine (3, 4, 47).

R29. In the event of a severe ADR, resumption of the infusion on the same day is not recommended, especially if symptoms persist or the cause of the event has not been identified (96% consensus).

In the event of suspected severe ADR, immediate discontinuation of the infusion is recommended (4). Unlike mild ADR, and despite clinical improvement after therapeutic measures, experts agree that administration should not be immediately restarted.

R30. Severe ADR associated with IVIG infusion, such as anaphylaxis, bronchospasm, and acute renal failure, require hospital management and a targeted evaluation in order to establish an appropriate subsequent therapeutic plan (100% consensus).

Anaphylaxis is a potentially life-threatening multisystem reaction that requires immediate attention in an emergency department, preferably in a resuscitation area. Initial management should follow the ABCDE approach, with continuous monitoring, high-flow oxygen, peripheral venous access, and blood sample collection. IM epinephrine is the first-line treatment, and early identification of airway obstruction and assessment for advanced support, including early intubation or vasopressors in refractory cases, are essential (48).

IVIG therapy may be associated with renal and electrolyte disturbances. Acute renal failure, although uncommon, may occur between 1 and 10 days after treatment initiation (49). Before prescribing IVIG, risk factors such as the use of nephrotoxic drugs, baseline renal function, age, diabetes mellitus, infusion rate, and the characteristics of the preparation used should be assessed (50, 51).

R31. In the event of a suspected anaphylactic reaction, IM epinephrine (1 mg/mL) should be administered immediately (96% consensus).

Epinephrine constitutes the first-line treatment for anaphylaxis and should be administered early (48). The IM route is the preferred route because of its rapid onset of action, using weight-adjusted doses in the pediatric population and 0.5 mg in adults, with repeat dosing according to clinical response if necessary (52, 53). IV epinephrine is reserved for patients with shock and requires close monitoring together with volume replacement (48).

**Figure 2:**
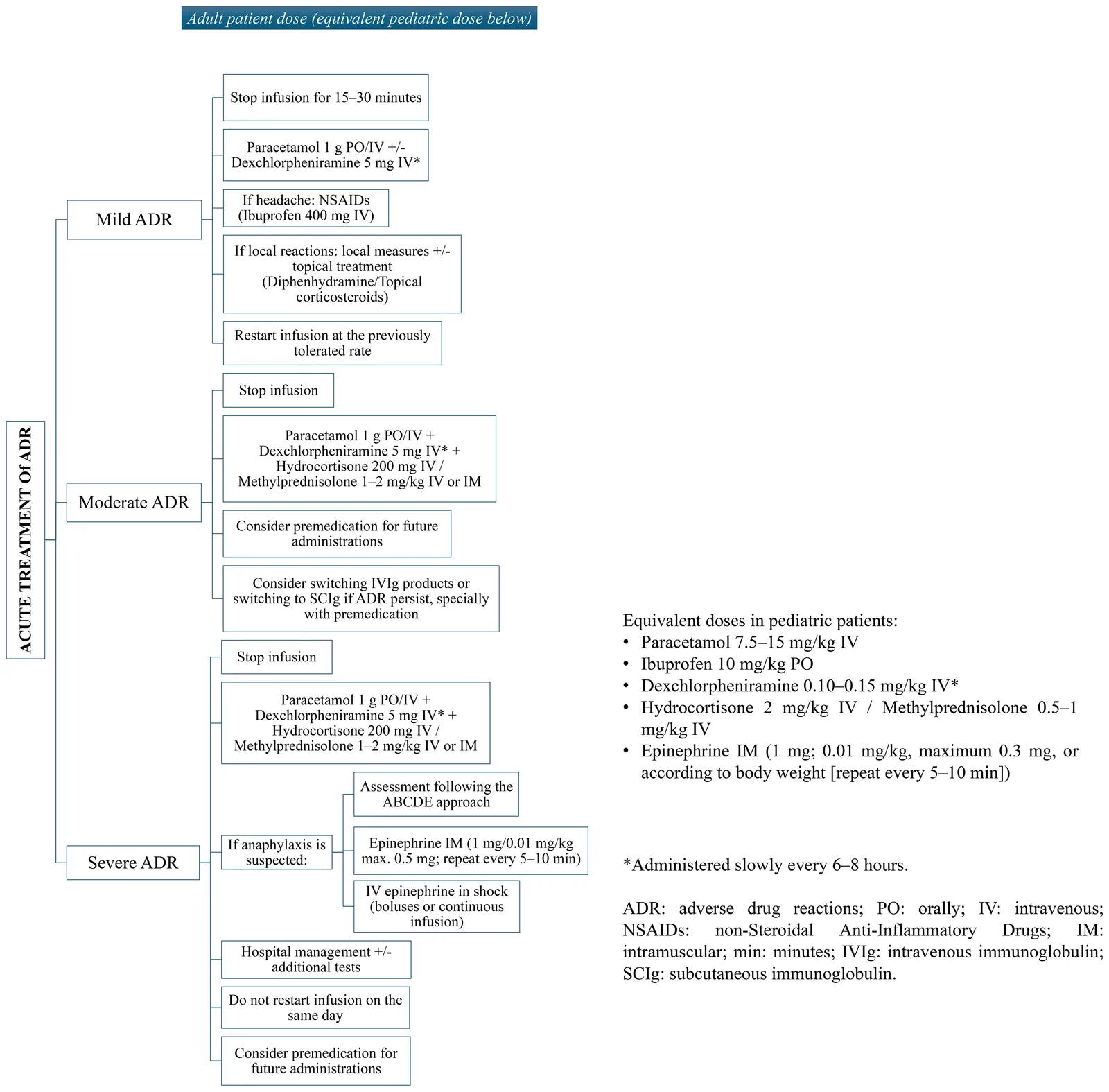
Practical algorithm for the acute treatment of ADR associated with IVIG/SCIG administration, stratified by severity. Stepwise therapeutic approach with recommended agents, doses, and routes of administration for adult and pediatric patients.

The acute management recommendations described in this section (R26–R31) are summarized in **Figure 2**, which presents a practical algorithm for the treatment of ADR stratified by severity. The algorithm specifies the stepwise therapeutic approach for mild, moderate, and severe ADR, including recommended agents, doses, and routes of administration for both adult and pediatric patients, and incorporates decision points such as infusion interruption, restart criteria, and indications for hospital transfer.

### 3.6 Complementary workup following ADR

R32. After a moderate or severe ADR during IVIG administration, it is advisable to perform at least basic laboratory studies such as complete blood count, creatinine, liver transaminases, erythrocyte sedimentation rate (ESR), and C-reactive protein (CRP) (80% consensus).

Early diagnosis and treatment of ADR associated with IVIG are essential to minimize complications. Among the recommended tests, a complete blood count may be useful for identifying hematological abnormalities such as hemolytic anemia or neutropenia described after IVIG infusion (2, 14, 54, 55).

Monitoring of renal function, including parameters such as serum creatinine, especially in the presence of risk factors or previous impairment, is indicated for the early detection of renal dysfunction (2, 54–57). Some experts have also recommended monitoring electrolyte levels, mainly to screen for hyponatremia and hyperkalemia (2).

Elevation of transaminases after IVIG administration is a frequent finding, generally mild and transient. Nevertheless, their determination may be useful in suspected ADR to detect abnormalities in liver profile or hepatic cytotoxicity (55).

CRP and ESR are nonspecific inflammatory markers that may be useful in the evaluation of moderate or severe ADR associated with IVIG (57). CRP shows a faster and more sensitive response in acute inflammatory processes, whereas ESR changes more slowly and may be influenced by non-inflammatory factors. Overall, after a moderate or severe ADR, it is recommended to perform basic studies such as complete blood count, creatinine, transaminases, CRP, and ESR, with additional tests added according to the clinical suspicion.

R33. In the event of an acute ADR to IVIG with symptoms suggestive of an allergic etiopathogenic mechanism (pruritus, erythema, wheals, etc.) or a reaction compatible with anaphylaxis, serum tryptase should be requested within the first 2–3 hours after the episode (88% consensus).

Tryptase is a specific marker of mast cell activation, and its transient elevation in serum supports the diagnosis of anaphylaxis (58, 59). Comparison of acute and baseline tryptase levels is recommended to confirm such activation; therefore, its determination may be useful in suspected immediate hypersensitivity reactions associated with IVIG.

### 3.7 Special considerations regarding anti-IgA antibodies

R34. In patients without a prior history of ADR, detection of anti-IgA antibodies should be interpreted within the clinical context and does not necessarily warrant modification of treatment or premedication (94% consensus).

The role of IgG anti-IgA antibodies in IVIG-associated anaphylaxis remains controversial. IgG anti-IgA antibodies can be detected in up to 40% of patients with selective IgA deficiency and in 9–25% of patients with common variable immunodeficiency, whereas IgE anti-IgA antibodies are much less frequent (60).

Some publications have associated IgG anti-IgA antibodies with the development of ADR, including non-IgE-mediated anaphylaxis, in patients with undetectable IgA levels (27, 61). However, current evidence has not demonstrated a clear causal association between their presence and an increased risk of severe reactions during IVIG administration, since some patients with these antibodies tolerate treatment, whereas others develop anaphylaxis in their absence (2, 20, 27, 62–65).

It is possible that IgG anti-IgA antibodies play a role in some rare ADR associated with IVIG (2, 20, 63–65), and they have been proposed as potential biomarkers of risk for non-IgE-mediated anaphylactoid reactions. Nevertheless, the available evidence is limited, and further studies are required to define their clinical relevance (62).

There are no unified recommendations regarding the indication and interpretation of IgG anti-IgA antibodies. Their usefulness in patients with low IgA levels without a history of transfusions or ADR has not been clearly established (63). Some reviews suggest determining these antibodies in patients with selective IgA deficiency and a history of reactions to blood products (66), as well as considering a switch to SCIG or the use of low-IgA-content preparations in patients with poor tolerance to IVIG (62). Overall, the available evidence suggests that the presence of anti-IgA antibodies alone may not be a consistent or reliable predictor of ADR to IG therapy. In the context of heterogeneous and sometimes conflicting data, routine modifications of treatment based solely on their detection may not be warranted in all patients. Therefore, clinical decisions should be individualized, considering the patient’s history of prior reactions and overall clinical context, rather than relying solely on the presence of anti-IgA antibodies.

R35. The choice of premedication should be based on the patient’s comorbidities and clinical history rather than on the presence or absence of serum IgA (94% consensus).

In this context, preventive strategies should also follow an individualized approach. Accordingly, the choice of premedication should be based on the patient’s comorbidities, prior infusion history, and overall clinical risk profile rather than solely on serum IgA levels. Given that anti-IgA antibodies are not consistently associated with ADR, their isolated detection does not necessarily justify routine premedication in otherwise asymptomatic patients (2, 4, 27, 62).

R36. In patients with undetectable serum IgA levels who experience a moderate or severe ADR, the determination of anti-IgA antibodies may be useful for etiological assessment and to guide the selection of preparations with lower IgA content or switching to SCIG for future infusions (75% consensus).

Although there is no consensus regarding the routine use of anti-IgA antibody testing in patients receiving IG therapy, their determination may be useful in selected cases. In patients with undetectable serum IgA levels who develop moderate or severe ADR, the assessment of anti-IgA antibodies can, in some cases, contribute to etiological clarification and support individualized management decisions, including the use of low-IgA content preparations or switching to SCIG for subsequent infusions (27, 62, 67).

R37. In patients with previous ADR and positivity for anti-IgA antibodies, serial determination has not demonstrated utility in clinical practice (92% consensus).

As discussed above, the available evidence on anti-IgA antibodies is limited beyond their initial identification. In addition, more recent data show no correlation between anti-IgA levels and the risk of transfusion reactions (66). Maintaining these findings and the overall limited evidence, it has not been demonstrated that repeated measurements provide additional clinically relevant information for clinical decision-making, particularly in the setting of ADR.

### 3.8 Special considerations regarding SCIG

R38. ADR associated with conventional or facilitated SCIG are mild in most cases and occur during the infusion or within the first hours afterward, usually in the form of erythema and pruritus at the infusion site (100% consensus).

ADR associated with SCIG, both conventional and facilitated, are frequent, although predominantly mild, local, and self-limited, consisting mainly of erythema, pruritus, edema, or discomfort at the infusion site, and are rarely associated with relevant systemic events (3, 68). Their frequency usually decreases over time, and they rarely lead to treatment discontinuation or are associated with severe systemic events (68, 69). The safety profile of facilitated SCIG is similar, with a predominance of mild local reactions and a very low incidence of severe ADR (70–72).

Transient SC nodules or indurations at the infusion site are also a recognized local reaction, generally benign and self-limited (73). Their frequency tends to decrease over time with adequate site rotation, technique optimization, and adjustment of needle depth and infusion volume per site.

R39. ADR such as headache, lower back pain, chills, asthenia, etc., are less common during SCIG infusion compared with those described with IVIG (100% consensus).

SCIG is associated with a lower frequency of systemic ADR than the IV route, with symptoms such as headache, fever, or chills being uncommon (3). This is related to its slow and sustained absorption, which avoids high serum peaks (74). In clinical practice, patients treated with SCIG show overall good tolerability, with a predominance of local reactions and absence of severe ADR (75).

R40. The most severe ADR associated with SCIG treatment, such as infection or cutaneous necrosis, are uncommon and usually occur at the administration site (100% consensus).

When more significant complications occur with the SC route, they are usually localized at the administration site. Reported cases of cutaneous necrosis reinforce that this type of event, in addition to being exceptional, is predominantly local (76, 77).

R41. Necrosis or other severe local reactions associated with SCIG administration are uncommon, and, in cases of cutaneous necrosis, maintenance of adequate local asepsis and application of topical antibiotic treatment, such as mupirocin or fusidic acid, should be indicated (92% consensus).

Severe ADR associated with the SC route are usually related to the infusion technique or tissue pressure at the puncture site and manifest as localized lesions (76, 78). In the event of suspected superinfection or loss of skin integrity, the use of topical antibiotics such as mupirocin or fusidic acid usually allows effective resolution, favoring a conservative and safe management approach for home therapy.

R42. Periodic re-education of patients and/or family members every 1–2 years is important to avoid administration errors or ADR associated with SCIG self-administration (100% consensus).

Home administration of SCIG requires that patients or caregivers receive adequate training and demonstrate competence in infusion technique and in the recognition of ADR (79). Clinical follow-up and periodic re-education, although without evidence supporting a specific interval, are essential to maintain safety, prevent errors, and promote adherence to treatment (71).

### 3.9 Statements that did not reach consensus

Two statements were evaluated across both Delphi rounds but did not reach the predefined consensus threshold of 75% agreement in either round and are therefore not endorsed as formal recommendations by the panel. They are included here for transparency and to inform future research and discussion.

Statement 1. In patients with no history of ADR or risk factors, IVIG treatment may be initiated without premedication, with subsequent assessment of its need based on tolerance (62.6% agreement).

The lack of consensus on this statement likely reflects differences in risk perception rather than disagreement with the principle of selective premedication endorsed elsewhere in this document. While the panel broadly supports a selective rather than universal approach to premedication (R10, R14, R18), some panelists expressed caution about omitting premedication specifically during the first infusion, which carries the highest risk of ADR in treatment-naïve patients (R3, R11). Thus, disagreement appears to center on the acceptable degree of caution at treatment initiation rather than on the overall premedication strategy.

Statement 2. Determination of interleukin 6 may be useful in patients experiencing a severe ADR, as it could contribute to the differential diagnosis of the underlying mechanisms (e.g., allergic reaction, cytokine release, etc.) (68.8% agreement).

The lack of consensus on this statement probably reflects the limited available evidence and the lack of standardization regarding the role of interleukin-6 in the evaluation of IG-associated ADR and its clinical utility. Although interleukin-6 may be useful in selected or complex cases, its routine use is limited by availability, lack of validated thresholds, and uncertain impact on management.

Both statements are considered relevant topics for future prospective studies and may be reconsidered in updated versions of these recommendations as new evidence becomes available.

## 4 Discussion

This Delphi-based consensus developed by the GISEI provides practical recommendations for the prevention, recognition, monitoring, and management of ADR associated with IV and SCIG therapy in patients with PID and SID. Although IG therapy has a well-established efficacy and favorable safety profile, ADR remains a clinically relevant issue that may affect treatment adherence, quality of life, and, in rare cases, patient safety (2–5). In this context, the present consensus addresses an important unmet need by providing clinically oriented recommendations focused specifically on infusion-related ADR in routine practice. In addition, practical algorithms for premedication and acute ADR management are provided to facilitate the implementation of the consensus recommendations in clinical practice.

One of the main findings of this study is the high level of agreement achieved for most recommendations, particularly those related to individualized risk assessment, infusion rate management, monitoring during administration, and early therapeutic intervention. These results reinforce the concept that most ADR associated with IG therapy are predictable, manageable, and potentially preventable through appropriate clinical protocols and experienced supervision (2–4, 15, 29, 42). In agreement with previous studies, the panel recognized infusion rate as one of the most important modifiable risk factors associated with ADR, supporting the recommendation to initiate IVIG infusions at the lowest possible rate, especially in treatment-naïve patients or after product changes (2–4, 15, 42).

The consensus also highlights the importance of patient-centered decision-making in IG therapy. The panel strongly agreed that the indication and administration strategy for IVIG or SCIG should be individualized according to patient comorbidities, previous tolerance, renal function, thrombotic risk, and treatment setting (2–4, 10, 11, 13). This recommendation is particularly relevant considering the heterogeneity of IG preparations, which differ in osmolality, stabilizers, sodium content, sugar composition, and residual IgA concentration, all of which may influence tolerability in susceptible patients (2, 3, 80). Although available evidence does not clearly demonstrate major differences in safety profiles among commercial products, maintaining treatment continuity with a well-tolerated preparation was considered preferable whenever possible (4, 43, 44, 80).

Another relevant aspect of the present consensus is the emphasis placed on structured monitoring and institutional protocols. The panel considered that centers administering IVIG regularly should implement standardized procedures, including documentation of infusion parameters, monitoring of vital signs, management algorithms for ADR, and clear premedication strategies (29, 30). This approach is consistent with current pharmacovigilance recommendations and may contribute to reducing practice variability between institutions. Furthermore, the recommendations underscore the importance of patient education before treatment initiation, particularly regarding recognition of post-infusion symptoms and indications for medical consultation (10–13). This aspect is especially relevant in the context of home-based SCIG administration.

Regarding premedication, the consensus supports a selective rather than universal approach. Interestingly, statements proposing systematic premedication for all patients did not achieve consensus, reflecting the limited evidence supporting routine prophylactic treatment in all individuals receiving IG therapy. Instead, the panel supported individualized premedication strategies based on previous ADR and patient risk factors. Paracetamol was considered the preferred first-line option for mild infusion-related symptoms, whereas antihistamines and corticosteroids were reserved for patients with prior moderate or severe ADR (3, 28, 31, 32, 39–41). These findings are aligned with current evidence suggesting that excessive premedication may not be necessary in most patients and should be balanced against potential ADR associated with corticosteroids or sedating antihistamines (3, 31, 39).

The present recommendations also reinforce the favorable safety profile of SCIG therapy. The panel consistently recognized that SCIG-associated ADR are predominantly mild, local, and self-limited, with a substantially lower frequency of systemic reactions compared with IVIG (3, 5, 68–72, 74, 75). This observation is consistent with the pharmacokinetic characteristics of SCIG, which produces slower and more stable IgG absorption and avoids the high serum peaks noticed after IVIG administration (5, 74). Consequently, SCIG was considered an appropriate therapeutic alternative in patients with poor IVIG tolerance (5, 45, 68, 69).

In addition, the consensus highlights the importance of periodic patient and caregiver re-education in order to minimize treatment-related errors, maintain confidence in self-administration, and optimize long-term adherence. This aspect is particularly relevant for SCIG therapy, as successful home-based treatment relies on patient empowerment, appropriate training, and active involvement in the management of therapy. Consistent with this concept, Pulvirenti et al. showed that the schedule and modality of IG administration significantly influence patients’ health-related quality of life (HR-QoL), underscoring the importance of individualized choice, patient involvement, and adequate training to optimize long-term management and adherence, regardless of the administration route (71, 81, 82).

The recommendations concerning anti-IgA antibodies deserve special consideration. Despite historical concerns regarding their association with anaphylactic reactions, current evidence has not demonstrated a clear causal association between their presence and an increased risk of severe ADR, as many patients with detectable antibodies tolerate IG therapy without complications, whereas severe reactions may also occur in their absence (60–67). Consequently, the panel agreed that clinical decisions should not be based solely on anti-IgA antibody detection but should instead consider the patient’s prior reaction history and overall clinical context. Premedication strategies should follow the same individualized approach, based on comorbidities and infusion history rather than on serum IgA levels alone. In selected cases, particularly patients with undetectable IgA who develop moderate or severe ADR, anti-IgA antibody testing may contribute to etiological assessment and guide the choice of low-IgA-content preparations or switching to SCIG. Finally, serial determination of these antibodies has not demonstrated clinical utility and is therefore not recommended in routine practice (67).

Beyond the evaluation of efficacy and safety outcomes, the assessment of HR-QoL provides important insights into the patient’s experience throughout long-term Ig replacement therapy. HR-QoL measures may help identify treatment-related issues that are not necessarily captured during the initial treatment period but may emerge over time. This is particularly relevant for patients receiving IVIG, in whom a “wear-off” effect has been described, with a decline in patient-reported well-being, increased susceptibility to infections, and worsening symptoms towards the end of the dosing interval. Rojavin et al. demonstrated that this phenomenon may occur during the final week of the IVIG cycle, highlighting the importance of incorporating patient-reported outcomes into the long-term monitoring and individualization of Ig replacement therapy (83).

This study has several strengths. First, it represents a nationwide multidisciplinary consensus involving clinicians with extensive experience in IG therapy across different clinical settings. Second, the Delphi methodology allowed anonymous evaluation of statements and facilitated convergence toward clinically meaningful recommendations. Third, the resulting document focuses specifically on practical aspects of ADR prevention and management, an area that is insufficiently addressed in many international IG therapy guidelines (6, 43, 81).

### 4.1 Limitations

However, several limitations should also be acknowledged. The recommendations are primarily based on expert opinion combined with heterogeneous evidence derived from observational studies, retrospective analyses, pharmacovigilance data, and clinical experience. High-quality randomized studies evaluating preventive strategies for IG-associated ADR remain limited. A formal grading system for the quality of evidence, such as GRADE, was not applied because the recommendations were primarily based on expert consensus in areas with limited and heterogeneous evidence. This should be considered when interpreting the strength of the recommendations.

In addition, two statements did not achieve consensus across both Delphi rounds, specifically those related to the initiation of IVIG without premedication in low-risk patients and the utility of interleukin-6 determination in the evaluation of severe ADR, highlighting areas where evidence remains insufficient or controversial. Furthermore, these recommendations were developed within the context of the Spanish healthcare system, and some aspects may vary according to local institutional protocols or product availability.

Nevertheless, many of the core principles outlined in this consensus are likely to be applicable across different clinical settings. Strategies based on individualized risk assessment, careful adjustment of infusion rates, early recognition and management of ADR, and patient education are broadly supported by the existing literature and are not inherently dependent on a specific healthcare structure. In contrast, certain aspects of implementation, such as premedication protocols, monitoring procedures, or the availability and selection of IG products (including switching practices and access to SCIG), may be influenced by local institutional protocols, national regulatory frameworks, and resource availability. Therefore, while these recommendations may serve as a practical framework for clinicians internationally, their application should be adapted to the specific organizational and healthcare context.

## 5 Conclusion

In conclusion, this Delphi-based consensus provides practical and clinically applicable recommendations for the management of ADR associated with IVIG and SCIG therapy in patients with PID and SID. The recommendations emphasize individualized treatment strategies, careful infusion management, appropriate monitoring, and early intervention in order to improve patient safety and treatment tolerability. Given the increasing use of IG therapy across multiple medical specialties and clinical settings, the implementation of standardized, evidence-based protocols may contribute to reducing variability in clinical practice and optimizing the quality of care. Future prospective studies are needed to further define risk factors, preventive measures, and biomarkers associated with severe ADR, as well as to evaluate the long-term impact of standardized management strategies on patient outcomes.

## Data Availability

The data supporting the conclusions of this article are available in the **Supplementary Material**.

## Supplementary material

Supplementary Table 1 Consensus recommendations on the prevention and management of ADR associated with IVIG/SCIG therapy: complete results of the modified Delphi consensus process, including all evaluated statements, levels of agreement, and consensus status.
