# Supplementary material for: Expert consensus recommendations for the prevention and management of immunoglobulin therapy-related adverse reactions in primary and secondary immunodeficiencies: a national Delphi study from the Immunodeficiency Group of the Spanish Society of Immunology
Source: Front Immunol. 2026 Aug 6;17:1901301. doi: 10.3389/fimmu.2026.1901301 (PMC13489857; doi:10.3389/fimmu.2026.1901301)
Supplement: Supplementary file 1 [file Table1.docx]

Supplementary Material

**Supplementary Table 1.** Consensus recommendations (R) on the management of ADR associated with IVIG and SCIG: results of the Delphi questionnaire.

| **Recommendations** | **Level of agreement** | **Consensus (Yes/No)** |
| --- | --- | --- |
| **1. General considerations regarding the administration of immunoglobulins** | | |
| R1. Before the administration of IVIG or SCIG, all patients must be informed about the characteristics of the treatment and possible ADR. They must receive clear instructions on when to contact the medical center if they experience post-infusion symptoms. | 96 | Yes |
| R2. The use of IVIG and SCIG should be assessed on an individualized basis for each patient, considering different factors, including comorbidities (renal failure, heart failure, hypertension, diabetes mellitus, etc.) | 96 | Yes |
| R3. In patients receiving IVIG for the first time, the infusion should always be started at the lowest possible rate, even when premedication has been administered. Subsequently, the rate may be increased gradually and in a controlled manner, according to the patient’s clinical tolerance and without ever exceeding the maximum recommended rate for the product. | 92 | Yes |

| **2. General considerations and classification of adverse reactions** | | |
| --- | --- | --- |
| R4. ADR such as facial erythema, fever, chills, asthenia, general malaise, mild headache, and pruritus are considered mild ADR. | 88 | Yes |
| R5. ADR such as urticaria, wheezing, myalgias, vomiting, chest pain, lower back pain, dizziness, arthralgias, nausea, hypertension, or severe headache are considered moderate ADR. | 84 | Yes |
| R6. ADR such as hypotension, bronchospasm, anaphylaxis, aseptic meningitis, thromboembolic events, acute renal failure, TRALI, or alterations in mental status are considered severe ADR. | 92 | Yes |
| R7. Most ADR to IVIG are mild, occur during the infusion or within the first 24 hours, and do not constitute a contraindication to continuing treatment. | 92 | Yes |
| R8. Moderate and severe ADR are uncommon or exceptional. | 96 | Yes |
| R9. Infusion rate, high dosage, higher concentration of the gammaglobulin preparation, and the absence of premedication are factors that may increase the risk of ADR. | 88 | Yes |

| **3. Premedication** | | |
| --- | --- | --- |
| R10. In patients initiating treatment with IVIG or SCIG, the indication for premedication should be individualized and based on clinical risk factors, medical history, and the care setting. | 94 | Yes |
| R11. In patients with a history of ADR, risk factors, or comorbidities that increase the likelihood of an ADR, premedication may be prescribed from the beginning. | 94 | Yes |
| R12. The use of premedication and monitoring of possible ADR during IVIG administration should be protocolized in hospital centers where it is administered regularly. | 88 | Yes |
| R13. Premedication should be administered at least 30 minutes before starting the IVIG infusion. | 88 | Yes |
| R14. Paracetamol (acetaminophen) is, in most cases, the first therapeutic step in patients requiring premedication before IVIG administration. | 79 | Yes |
| R15. The recommended dose of paracetamol as premedication is 1 gram, administered orally or intravenously. | 88 | Yes |
| R16. Premedication with H1 antihistamines (such as dexchlorpheniramine or diphenhydramine) is recommended in patients with a history of rash, pruritus, or urticaria associated with previous IVIG administration. | 84 | Yes |
| R17. The recommended premedication dose of dexchlorpheniramine is 5 mg IV (or the equivalent according to the specific drug or the corresponding weight-based dose in children)***.*** | 88 | Yes |
| R18. Premedication with corticosteroids should be reserved for cases with previous moderate or severe ADR. | 92 | Yes |
| R19. For corticosteroid premedication, hydrocortisone 75 mg, dexamethasone 4 mg IV, or methylprednisolone 40 mg IV are preferred, or the corresponding weight-based doses in children. | 88 | Yes |
| R20. In patients with risk factors or a history of moderate or severe ADR, premedication with combinations of paracetamol, H1 antihistamines, and corticosteroids is preferable to monotherapy. | 84 | Yes |
| R21. The need for premedication and the drugs used should be adjusted according to the patient's tolerance and may be progressively reduced until completely withdrawn if no associated risk factors are present. | 88 | Yes |

| **4. General management of adverse reactions** | | |
| --- | --- | --- |
| R22. Although there are no major differences in IVIG-associated ADR among different brands or formulations, switching products without a justified reason is not recommended in order to avoid sensitization. | 80 | Yes |
| R23. Switching IVIG preparations should be reserved for patients with previous ADR. | 84 | Yes |
| R24. In patients who do not tolerate the administration of any IVIG preparation, the use of SCIG is a good therapeutic option to maintain treatment. | 96 | Yes |
| R25. Monitoring during the first two hours of infusion should be maintained even if the patient has received premedication. | 96 | Yes |

| **5. Immediate treatment of adverse reactions** | | |
| --- | --- | --- |
| R26. Reducing the infusion rate constitutes the first measure in the management of mild ADR. | 92 | Yes |
| R27. Treatment with paracetamol and/or antihistamines constitutes the first therapeutic step in mild ADR and, in most cases, is sufficient. | 100 | Yes |
| R28. In the event of a severe ADR to IVIG infusion, administration should be stopped immediately, adequate IV hydration should be ensured, and symptomatic treatment should be initiated, which may include corticosteroids, antihistamines, analgesics, or epinephrine, as indicated. In addition, transfer or admission to a hospital should be considered depending on the patient’s clinical course. | 100 | Yes |
| R29. In the event of a severe ADR, resumption of the infusion on the same day is not recommended, especially if symptoms persist or the cause of the event has not been identified. | 96 | Yes |
| R30. Severe ADR associated with IVIG infusion, such as anaphylaxis, bronchospasm, and acute renal failure, require hospital management and a targeted evaluation in order to establish an appropriate subsequent therapeutic plan. | 100 | Yes |
| R31. In the event of a suspected anaphylactic reaction, IM epinephrine (1 mg/mL) should be administered immediately. | 96 | Yes |

| **6. Complementary workup following ADR** | | |
| --- | --- | --- |
| R32. After a moderate or severe ADR during IVIG administration, it is advisable to perform at least basic laboratory studies such as complete blood count, creatinine, liver transaminases, ESR, and CRP***.*** | 80 | Yes |
| R33. In the event of an acute ADR to IVIG with symptoms suggestive of an allergic etiopathogenic mechanism (pruritus, erythema, wheals, etc.) or a reaction compatible with anaphylaxis, serum tryptase should be requested within the first 2–3 hours after the episode. | 88 | Yes |

| **7. Special considerations regarding anti-IgA antibodies** | | |
| --- | --- | --- |
| R34. In patients without a prior history of ADR, detection of anti-IgA antibodies should be interpreted within the clinical context and does not necessarily warrant modification of treatment or premedication. | 94 | Yes |
| R35. The choice of premedication should be based on the patient’s comorbidities and clinical history rather than on the presence or absence of serum IgA. | 94 | Yes |
| R37. In patients with previous ADR and positivity for anti-IgA antibodies, serial determination has not demonstrated utility in clinical practice. | 92 | Yes |

| **8. Special considerations regarding subcutaneous immunoglobulins** | | |
| --- | --- | --- |
| R38. ADR associated with conventional or facilitated SCIG are mild in most cases and occur during the infusion or within the first hours afterward, usually in the form of erythema and pruritus at the infusion site. | 100 | Yes |
| R39. ADR such as headache, lower back pain, chills, asthenia, etc., are less common during SCIG infusion compared with those described with IVIG***.*** | 100 | Yes |
| R40. The most severe ADR associated with SCIG treatment, such as infection or cutaneous necrosis, are uncommon and usually occur at the administration site. | 100 | Yes |
| R41. Necrosis or other severe local reactions associated with SCIG administration are uncommon, and, in cases of cutaneous necrosis, maintenance of adequate local asepsis and application of topical antibiotic treatment, such as mupirocin or fusidic acid, should be indicated. | 92 | Yes |
| R42. Periodic re-education of patients and/or family members every 1–2 years is important to avoid administration errors or ADR associated with SCIG self-administration. | 100 | Yes |

ADR: adverse drug reactions; IG: immunoglobulins; IVIG: intravenous immunoglobulin; SCIG: subcutaneous immunoglobulin; IM: intramuscular; TRALI: transfusion-related acute lung injury; CRP: C-reactive protein; ESR: erythrocyte sedimentation rate.
